# Cost-Effectiveness of Angiotensin-Converting Enzyme Inhibitors for the Prevention of Diabetic Nephropathy in The Netherlands – A Markov Model

**DOI:** 10.1371/journal.pone.0026139

**Published:** 2011-10-11

**Authors:** Charles Christian Adarkwah, Afschin Gandjour, Maren Akkerman, Silvia M. Evers

**Affiliations:** 1 CAPHRI School of Public Health and Primary Care, Maastricht University, Maastricht, The Netherlands; 2 Department of Medicine III, RWTH-University Hospital Aachen, Aachen, Germany; 3 Frankfurt School of Finance & Management, Frankfurt, Germany; 4 Faculty of Medicine, RWTH-University Aachen, Aachen, Germany; Federal University of São Paulo, United States of America

## Abstract

**Objective:**

Type 2 diabetes is the main cause of end-stage renal disease (ESRD) in Europe and the USA. Angiotensin-converting enzyme (ACE) inhibitors have a potential to slow down the progression of renal disease and therefore provide a renal-protective effect. The aim of our study was to assess the most cost-effective time to start an ACE inhibitor (or an angiotensin II receptor blocker [ARB] if coughing as a side effect occurs) in patients with newly diagnosed type 2 diabetes in The Netherlands.

**Methods:**

A lifetime Markov decision model with simulated 50-year-old patients with newly diagnosed diabetes mellitus was developed using published data on costs and health outcomes and simulating the progression of renal disease. A health insurance perspective was adopted. Three strategies were compared: treating all patients at the time of diagnosing type 2 diabetes, screening for microalbuminuria, and screening for macroalbuminuria.

**Results:**

In the base-case analysis, the treat-all strategy is associated with the lowest costs and highest benefit and therefore dominates screening both for macroalbuminuria and microalbuminuria. A multivariate sensitivity analysis shows that the probability of savings is 70%.

**Conclusions:**

In The Netherlands for patients with type 2 diabetes prescription of an ACE inhibitor immediately after diagnosis should be considered if they do not have contraindications. An ARB should be considered for those patients developing a dry cough under ACE inhibitor therapy. The potential for cost savings would be even larger if the prevention of cardiovascular events were considered.

## Introduction

The prevalence of type 2 diabetes and its secondary complications will rise [1]–[3] due to ageing population and growing obesity. This type of diabetes represents the most common form of carbohydrate disorders affecting at least 5% of the population in the industrialized world [4]. As a result higher costs for diabetes treatment in general and especially treatment of secondary complications will be a huge burden for health care systems. Type 2 diabetes is the main cause of end-stage renal disease (ESRD) in the Netherlands [5] as well as in other European countries and the United States [6]–[7]. Diabetic nephropathy leads to a gradual decline of the renal function and is initially characterized by micro- or macroalbuminuria. Diabetic nephropathy may progress to ESRD, which is defined by the need for either long-term dialysis or renal transplantation [8]. The prevalence of patients in renal replacement therapy in the Netherlands doubled within the last 15 years [9]. In 2010, about 15 000 patients underwent renal-replacement therapy. In the last five years, the proportion of transplanted patients has been continuously increasing and represents about 57% of all patients requiring renal replacement therapy [9].

The costs of ESRD treatment are rather high, with a share of the national expenditures in European countries ranging from 0.7% in the UK to 1.8% in Belgium [10], [11], with a share in the Netherlands of about 1.3%. In the Netherlands, the costs of ESRD treatment amount to €42 000 per patient per year [10], [12], [13]. Hence, prevention of ESRD is not only important from a medical, but also from an economic viewpoint.

Angiotensin converting enzyme (ACE) inhibitors slow down the progression of diabetic nephropathy independent of an elevated blood pressure [14], [15]. Angiotensin receptor blockers (ARBs) have similar effects on renal outcomes in diabetic patients [16] but are more expensive, mostly due to patent protection. Evidence suggests that the only major clinical difference between these classes of drugs is a higher risk of dry cough associated with ACE inhibitors [17].

Several national and international clinical practice guidelines recommend starting ACE inhibitor therapy in diabetic patients with (micro)albuminuria [18]-[20]. However, physician compliance in the Netherlands as well as in many other European countries is rather low [21]. Cost-effectiveness models conducted in the United States by Golan et al. (1999) [22], Rosen et al. (2005) [23] and in Germany by Adarkwah et al. (2010) [24] suggest that the best starting point for ACE inhibitor therapy is immediately after diagnosis of diabetes. For the Netherlands no data are available on the cost-effectiveness of ACE inhibitor therapy in diabetic patients with (micro)albuminuria. However, results of the non-Dutch studies may not be transferable to the Netherlands. Transferability of economic evaluation studies between countries is hindered by a number of factors such as demography, the epidemiology of the disease, availability of health care resources and differences in reimbursement systems between countries, in particularly due to variances in absolute and relative costs/prices.

The goal of this study is to present a cost-effectiveness model, which determines the best time to start an ACE inhibitor in newly diagnosed patients with type 2 diabetes and without hypertension or heart failure in the Netherlands. The analysis is conducted from a health care perspective in order to increase comparability to other models on this topic [22]-[24]. In our model we included ARBs as an alternative for patients who experience ACE-inhibitor-induced cough. In the base case the age of 50 years was assumed as the mean age of diagnosing type 2 diabetes [25], [26].

## Methods

### Overview and Model Design

**Figure 1:**
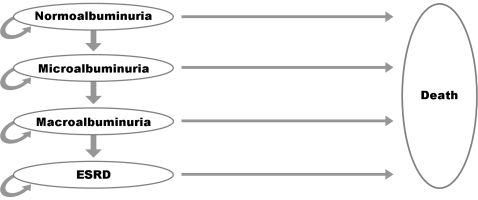
Schematic representation of the Markov decision model.

Is it cost-effective to treat all newly diagnosed type 2 diabetic patients in the Netherlands with an ACE inhibitor to prevent renal disease? We conducted a cost-utility analysis and measured health outcomes in terms of quality-adjusted life years (QALYs). We adapted a Markov decision model previously developed for the German setting [24] and also proven applicable for non-diabetic advanced renal disease [27] in order to simulate the course of a cohort of 1 000 patients at the age of 50 years as it progresses to microalbuminuria, macroalbuminuria, ESRD, and death. A Markov model is an iterative process where patients are assumed to stay in one cycle (i.e., a defined health state) for a certain time and then make a transition to another cycle. Markov models are useful when a decision problem involves risk that is continuous over time, when the timing of events is important, and when important events may happen more than once. The model was built in Microsoft Excel® 2007. We chose a cycle length of one year for the health states defined by the Markov model because all transition probabilities gathered from the literature referred to a duration of at least one year. All input data included in the model can be found in table 1. Our Markov model contains the following five health states (Figure 1), which represent the occurrence of events after model entry:

1. type 2 diabetes, with normoalbuminuria (excretion < 30 mg/d)
2. type 2 diabetes, with microalbuminuria (excretion 30–300 mg/d)
3. type 2 diabetes, with macroalbuminuria (excretion >300 mg/d)
4. ESRD (treated with dialysis or renal transplantation)
5. death

**Table 1:** Data used to determine the cost-effectiveness of ACE inhibitors and ARBs in newly diagnosed type 2 diabetes.

Variable	Base-case estimate	Range tested*	Reference
**Initial disease prevalence, %**			
Normoalbuminuria	79	66.5–100	[28]
Microalbuminuria	18	0–27.6	[28]
Macroalbuminuria	3	0–5.9	[28]
**Annual transition probabilities (without ACE inhibitors)**			
Normoalbuminuria to microalbuminuria	0.056	0.03–0.08	[29]
Microalbuminuria to macroalbuminuria	0.094	-0.02–0.20	[30]
Macroalbuminuria to ESRD	0.056	0.025–0.08	[15]
Normo-/micro-/macro-albuminuria to death	Age-dependent	–	[35]
ESRD to death	0.09	–	[9]
**Relative risk for progression with ACE inhibitors**			
Normoalbuminuria to microalbuminuria	0.60	0.43–0.84	[29]
Microalbuminuria to macroalbuminuria	0.45	0.29–0.69	[30]
Macroalbuminuria to ESRD	0.61	0.50–0.75	[15]
**Utilities (health states)**			
Diabetes (baseline health)	0.88	0.86–0.90	[38]
ESRD	0.62	0.39–0.84	[39]
**ACE inhibitor/ARB treatment**	1.00	0.95–1.00	[63]
**Annual costs, €**			
General health care expenditures	3.310,23 -23.626,23 (age-dependent)	–	[43], [44], [55]
Per-patient cost of diabetes compared to non-diabetic population	547	–	[43], [44], [55]applied to all health states except for ESRD
ACE inhibitor (20mg enalapril daily)	6.96	–	[46]applied to all health states except for ESRD
ARB (300mg irbesartan daily)	298.68	–	[46], [47]applied to all health states except for ESRD
Mixed drug therapy costs (9.9% treated with ARBs)	62.70	62.70–83.78	[46]applied to all health states except for ESRD
Screening for microalbuminuria	7.00	–	[58], [59]
Screening for macroalbuminuria	1.12	–	[58], [59]
ESRD	42 110	33 688–50 532	[9], [13]
Transplantation	14 387	–	[9], [13]
Dialysis	79 112	–	[9], [13]
Home/in-center hemodialysis	83 217	–	[9], [13]
Continuous ambulatoryperitoneal dialysis	54 067	–	[9], [13]
Continuous cyclingperitoneal dialysis	69 546	–	[9], [13]
**SMR**	1.41	1.39–1.43	[1]
**Rate of ARB use, %**	9.9	9.6–10.2	[17]
**Specificity of HPLC** (microalbuminuria screening procedure)	1.00	0.81–1.00	[53]
**Discount rate of costs**	0.04	0.00–0.10	[44], [54], [55];
**Discount rate of benefits**	0.015	0.00–0.10	[44], [54], [55];

ACE  =  angiotensin-converting enzyme; ARB  =  angiotensin II receptor blocker; ESRD  =  end-stage renal disease; HPLC  =  high performance liquid chromatography; SMR  =  standardized mortality ratio.

We assumed that diabetic nephropathy progresses without skipping any stage. Further, patients may die at any time (stage 5). The states of albuminuria were defined according to the recommendations of the American diabetes Association [20]. During each cycle, patients accumulate utility (measured by QALYs) and costs. A half-cycle correction was applied to both costs and outcomes to allow for transition events occurring mid-way through each 12-month cycle.

The simulation was done until the age of 99. Hence, the time horizon is 50 years. The age of 99 was chosen as a cutting point as there are no mortality data available beyond this age. Regardless, more than 99% of patients in the simulation are dead at this age.

### Clinical Strategies

Three starting points for ACE inhibitors were considered [3], [22]. In the “screen for microalbuminuria” strategy patients are screened for microalbuminuria once a year and treatment is started if the test result is positive. In the “screen for macroalbuminuria” strategy patients are screened for macroalbuminuria once a year and treatment is also started if the test result is positive. In the “treat all” strategy no screening is performed at all and patients start on ACE inhibitor therapy at the time of diagnosing type 2 diabetes. In addition, the analysis performed included the ARB option for the entire patient population in all three strategies reflecting a more expensive treatment. To find information on the distribution of health states at the time of diagnosis, we used the following search strategy in the PubMed database (date: February 08, 2011): *(newly diagnosed[All Fields]) AND macroalbuminuria[All Fields] AND microalbuminuria[All Fields] AND prevalence[All Fields] AND (albumin excretion [All Fields]) NOT (type 1 diabetes [All Fields])*. We obtained 2 hits. Thereof one study was excluded because it was conducted among Pima Indians. The other one is a Finnish prospective observational study [28], which was conducted from 1982 to 1992. In this study, the distribution of health states at the time of diagnosis (average age: 58 years) was as follows: 79% normoalbuminuria, 18% microalbuminuria, and 3% macroalbuminuria. We tested the impact of the initial distribution on results in a sensitivity analysis.

### Transition Probabilities

In order to identify studies on the effectiveness of ACE inhibitor or ARB therapy on the prevention of diabetic kidney disease we searched in the Cochrane Database of Systematic Reviews using the search strategy *normoalbuminuria OR microalbuminuria OR macroalbuminuria*. We found two meta-analyses proving evidence that ACE inhibitors halt the transition from normo- to microalbuminuria and micro- to macroalbuminuria [29], [30]. These meta-analyses pooled studies on patients with type 1 and type 2 diabetes, as heterogeneity did not appear to an issue. Compared to placebo, ACE inhibitors significantly reduced the development of microalbuminuria (six trials, 3 840 patients: relative risk (RR) 0.60, 95% confidence interval (CI) 0.43 to 0.84), and the progression from microalbuminuria to macroalbuminuria (17 trials, 2 036 patients: RR 0.45, 95% CI 0.29 to 0.69).

In order to identify studies on the effectiveness of ACE inhibitors or ARBs on the transition from macroalbuminuria to ESRD, evidence-based clinical practice guidelines were checked on the prevention of diabetic nephropathy [19], [31]–[33]. One randomized clinical trial [15] was identified that was rated as well-designed randomized controlled trial (RCT) [19], [33] providing high-grade evidence. In this trial captopril significantly reduced the development of ESRD compared to placebo (409 patients, RR 0.61, 95% CI 0.50 to 0.75).

To determine annual transition probabilities we first calculated a total probability for each arm, by dividing the number of events (ESRD) during the trial period by the number of patients. Next, we determined annual transition probabilities by assuming a constant annual hazard rate over the study time horizon [34]. A constant hazard rate yields an exponential survival curve.

In patients with normo-, micro-, and macroalbuminuria mortality is a function of age and was calculated by multiplying age-specific mortality rates of the Dutch general population [35] with a standardized mortality ratio for patients with diabetes compared to the general population [1], [36]. For patients with normo-, micro-, and macroalbuminuria we assumed that mortality is stage-independent as there are no valid data showing that a significant difference exists. For patients with ESRD, we calculated the annual mortality rate based on 13 905 patients in the Netherlands [9], by dividing the annual number of decedents by the total number of patients. While the annual number of decedents treated with dialysis could be derived from the website, the number of decedents with a transplant was obtained by personal communication (A. Hemke, Dutch End-Stage Renal Disease Registry, March 17, 2011).

### Preference Weights

We included preference weights of diabetic patients (table 1) from a published cross-sectional study [37]. Adult diabetic patients (n  =  292) with a disease duration of at least one year and a mean age of 62 years (range 21–85) were interviewed by the time trade-off (TTO) method. We assumed that patients with normo-, micro-, or macroalbuminuria do not suffer from an additional reduction in health-related quality of life [38]. There is no convincing evidence in the literature that confirms a utility decrease merely due to albuminuria. The preference weight for ESRD was taken from a systematic review of empirical studies in which TTO weights were provided by patients [39]. The TTO is the most commonly used method to elicit quality-of-life weights for QALYs [40], [41]. The TTO technique determines the proportion of remaining life years in poor health patients are willing to give up or trade in exchange for perfect health. Based on patient responses utility scores are calculated. Utility measures in economic evaluations are becoming increasingly important given the fact that decision makers are asked to optimize the allocation of scarce health care resources across disease areas and patient groups [42]. Values are similar to EQ-5D scores (baseline value 0.61) reported by de Wit et al. (1998) [13].

### Costs

As stated, the analysis is conducted from the health care perspective. Hence, only direct costs and direct health effects – defined as life years gained – were considered. Costs were inflated to year 2010 euros using data on the consumer price index [43]. Costs of ACE inhibitors, ARBs, annual screening procedures, and treatment for ESRD as well as health care expenditures related and unrelated to diabetes were taken into account. The recommendations of the Dutch guidelines for pharmacoeconomic research were followed [44]. For ACE inhibitor therapy the most frequently prescribed ACE inhibitor in the Netherlands, enalapril, [45] was taken into consideration. In the base case, the cheapest generic of enalapril 10 mg daily was used, whereas the most expensive one was applied in the sensitivity analysis [46]. For ARBs we considered a dose of 300 mg irbesartan daily [46], which is more effective in renal protection than a dose of 150 mg [47]. The costs of enalapril and irbesartan treatment were based on 2011 Dutch prices and include 6% value-added tax as well as a 3-monthly pharmacists' prescription fee of €7.50 [48]. As recommended by a published health technology assessment (HTA) report [49] and a national clinical chemistry report [50], a quantitative screening test for microalbuminuria (high performance liquid chromatography or immunoturbidimetrie) was preferred over a semi quantitative one (e.g., Micral-Test®) [51], [52], because it demonstrates higher sensitivity (100%) [52] and specificity (81-98%) [53]. Bakker et al. [51] clearly state that a simple dipstick test is not sufficient to detect microalbuminuria at an early stage. In the base-case analysis we assumed a specificity of 100% which is conservative because treating false positives (i.e., patients with normoalbuminuria) leads to cost savings. In the sensitivity analysis we applied a specificity of 81%. To screen for macroalbuminuria we used a dipstick test applied in a general practitioner's office recommended by the Dutch Kidney Check Campaign [31], [49].

The annual costs of patients with ESRD were calculated as a weighted average of the costs of different types of dialysis as well as renal transplantation based on a Dutch study [13] and prevalence data available from the national register [9].

In detail, the following calculations were made (see table 2 in the appendix for details):

1. cost of dialysis  =  *β*_1_ *x*_1_ + *β*_2_ *x*_2_ + *β*_3_ *x*_3_  =  X, where *x*_n_  =  annual cost of dialysis treatment *n  = * 1,2,3; *β*_n_  =  prevalence weight of the dialysis treatment, and *β*_1_ + *β*_2_ + *β*_3_  =  1
2. cost of ESRD  =  *p*X + (1-*p*)Y, where Y  =  cost of renal transplantation and *p*  =  proportion of ESRD treated by dialysis treatment.

**Table 2:** Parameters used for calculating the cost of end-stage renal disease (see cost section under “Methods”).

variable	meaning
1	home/center hemodialysis
2	continuous ambulatory peritoneal dialysis (CAPD)
3	continuous cycling peritoneal dialysis (CCPD
*β*_1_	0.82
*β*_2_	0.106
*β*_3_	0.074
*x*_1_	€ 83 217
*x*_2_	€ 54 067
*x*_3_	€ 69 546
p	0.43
X	€ 79 112
Y	€ 14 387

A transplant survival of 10 years was assumed and a distinction made between the first year of transplantation and the years following. Costs were inflated to 2010 Dutch prices.

Health care expenditures related and unrelated to diabetes were both included. Costs were discounted at an annual rate of 4% whereas benefits were discounted at an annual rate of 1.5% in accordance with the CVZ recommendations [44], [54], [55].

### Sensitivity Analyses

To address uncertainty around mean incremental costs and effectiveness, univariate sensitivity analyses were conducted. Whenever possible, we run the analysis using the upper and lower bound of the 95% CI of the mean.

In order to assess how a simultaneous change of several variables affects the incremental cost-effectiveness ratio (ICER), we performed a Monte Carlo simulation, a type of multivariate sensitivity analysis. This technique runs a large number of simulations (here: 1 000) by repeatedly drawing samples from probability distributions of input variables. Thus, it provides a probability distribution for the output variables, i.e., incremental costs and effectiveness. Probabilities and relative risks were assumed to follow a beta distribution Beta(α, β) because they are restricted to take on values between 0 and 1. Because the distribution of health states at the time of diagnosis had more than 2 outcomes, we assumed a Dirichlet distribution Dirichlet(α1, α2, . . ., αk) [37]. Cost data were assumed to follow a gamma distribution Gamma(a, b) because they are normally distributed but restricted to take on values between 0 and 1. The standard deviation of probabilities and relative risks was calculated according to the following formula [56]:
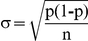



Given the ambiguous interpretation of negative ICERs, we transformed ICERs into net monetary benefits (NMBs). We generated a cost-effectiveness acceptability curve based on the distribution of NMBs for each value of the willingness to pay per QALY gained. A cost-effectiveness acceptability curve allows a decision maker to consider whether a prevention strategy is cost-effective in relation to the maximum amount a decision-maker is willing to pay for a QALY. At each ceiling value for the willingness to pay for a QALY, the cost-effectiveness acceptability curve shows the probability that treatment is cost-effective. The input data for the model are summarized in table 1.

## Results

### Base-case Analysis

The base-case analysis, which applies to 50-year-old patients, shows that “no screening and treatment”, “screening for macroalbuminuria”, and “screening for microalbuminuria” are all dominated by the “treat all” strategy, which is associated with the lowest costs and highest benefit (table 3). Again, the “treat all” strategy implies that all patients are treated with an ACE inhibitor (or an ARB in the event of cough).

**Table 3:** Results of the base-case analysis, based on mean estimates of input variables.

Strategy	Costs (€)	Undiscounted LYs	Discounted QALYs	ICER (€/QALY)
Screening for macroalbuminuria	110 777	28.52	19.15	dominated
Screening for microalbuminuria	101 140	28.88	19.54	dominated
Treating all patients with ACEIs/ARBs	98 421	28.94	19.63	dominant

ACEI  =  angiotensin-converting enzyme inhibitor; ARB  =  angiotensin II receptor blocker; LYs  =  life years; QALY  =  quality-adjusted life-years; ICER  =  incremental cost-effectiveness ratio.

### Sensitivity Analysis

In the univariate sensitivity analysis, variables with the largest impact on incremental costs and effectiveness are the absolute risk for progression from micro- to macroalbuminuria without ACE inhibition as well as the relative risk for progression from normo- to microalbuminuria with ACE inhibitor therapy and the discount rate (see table 4 for details). When assuming a low progression rate from micro- to macroalbuminuria without ACE inhibition, screening for microalbuminuria dominates the “treat all” strategy. A threshold sensitivity analysis shows that at an annual drug cost of €426.70 (base case: €62.70) the breakeven point is reached. The probability of savings is 70%.

**Table 4:** Univariate sensitivity analyses: effects of varying base-case estimates on the incremental cost-effectiveness ratio of treating all patients with ACE inhibitors vs screening for microalbuminuria (reference strategy).

	Incremental costs	Incremental QALYs	Incremental cost-effectiveness ratio
**Initial disease prevalence: Proportion of normoalbuminuric patients, %**			
Lower bound	−2 289	0.080	−28 647
Higher bound	−3 442	0.120	−28 647
**Annual transition probabilities (without ACE inhibitors)**			
**from normo- to microalbuminuria**			
Lower bound	-1 712	0.062	−27 659
Higher bound	−3 348	0.123	−27 214
**from micro- to macroalbuminuria**			
Lower bound	1 238	-0.22	−57 155
Higher bound	−4 604	0.166	−27 736
**from macroalbuminuria to ESRD**			
Lower bound	−1 202	0.047	−25 823
Higher bound	−3 625	0.126	−28 661
**Relative risk for progression with ACE inhibitors**			
**from normo- to microalbuminuria**			
Lower bound	−4 352	0.141	−30 831
Higher bound	−734	0.036	−20 510
**from micro- to macroalbuminuria**			
Lower bound	−1 836	0.066	−27 921
Higher bound	− 3 730	0.131	-28 403
**from macroalbuminuria to ESRD**			
Lower bound	−2 274	0.080	-28 358
Higher bound	−3 229	0.112	−28 727
**Utilities (health states)**			
**Diabetes (baseline health)**			
Lower bound	−2 719	0.090	−30 264
Higher bound	−2 719	0.100	−27 194
**ESRD**			
Lower bound	−2 719	0.142	−19 081
Higher bound	−2 719	0.049	−55 041
**Disutility of ACE inhibitor treatment**			
Lower bound	−2 719	0.092	−29 554
Higher bound	−2 719	0.095	−28 647
**Costs**			
**ACE inhibitor**			
Lower bound	−2 719	0.095	−28 647
Higher bound	−2 569	0.095	-27 062
**ESRD**			
Lower bound	−1 858	0.095	−19 581
Higher bound	−3 579	0.095	−37 713
**SMR**			
Lower bound	−2 723	0.096	−28 249
Higher bound	−2 715	0.093	−29 046
**Rate of ARB use**			
Lower bound	−2 419	0.095	−25 463
Higher bound	−2 854	0.095	−30 042
**Specificity of HPLC (microalbuminuria screening procedure)**			
81%	− 1 853	0.039	−47 513
**Discount rate of costs**			
0%	−9 179	0.095	−96 710
1.5%	−5 708	0.095	−60 140
4%	−2 719	0.095	−28 647
7%	−1 189	0.095	−12 523
10%	−537	0.095	−5 655
**Discount rate of effects**			
0%	−2 719	0.139	−19 592
1,5%	−2 719	0.095	−28 647
4%	−2 719	0.051	−52 850
7%	−2 719	0.026	−105 670
10%	−2 719	0.014	−200 909

QALYs  =  quality-adjusted life years; ACE  =  angiotensin-converting enzyme; ESRD  =  end-stage renal disease;

SMR  =  standardized mortality ratio; HPLC  =  high performance liquid chromatography

“Lower bound” and “higher bound” refer to the limits of the 95% confidence interval.

**Figure 2:**
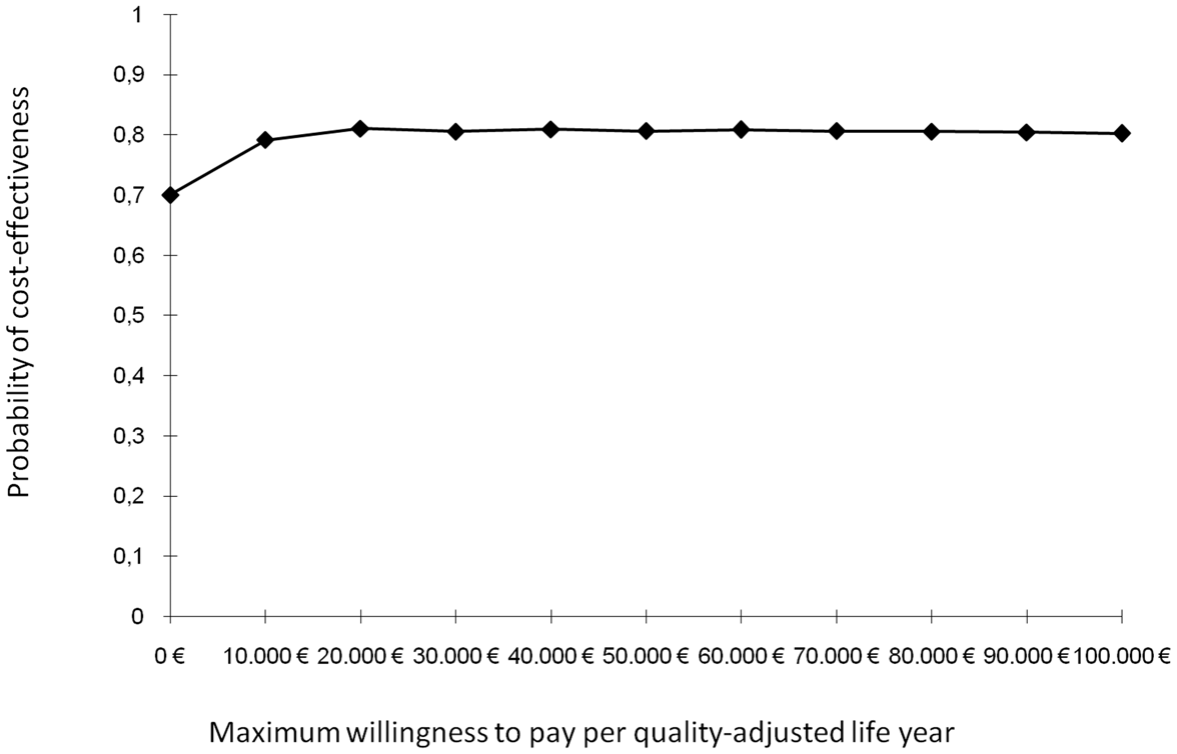
Cost-effectiveness acceptability curve.

**Figure 3:**
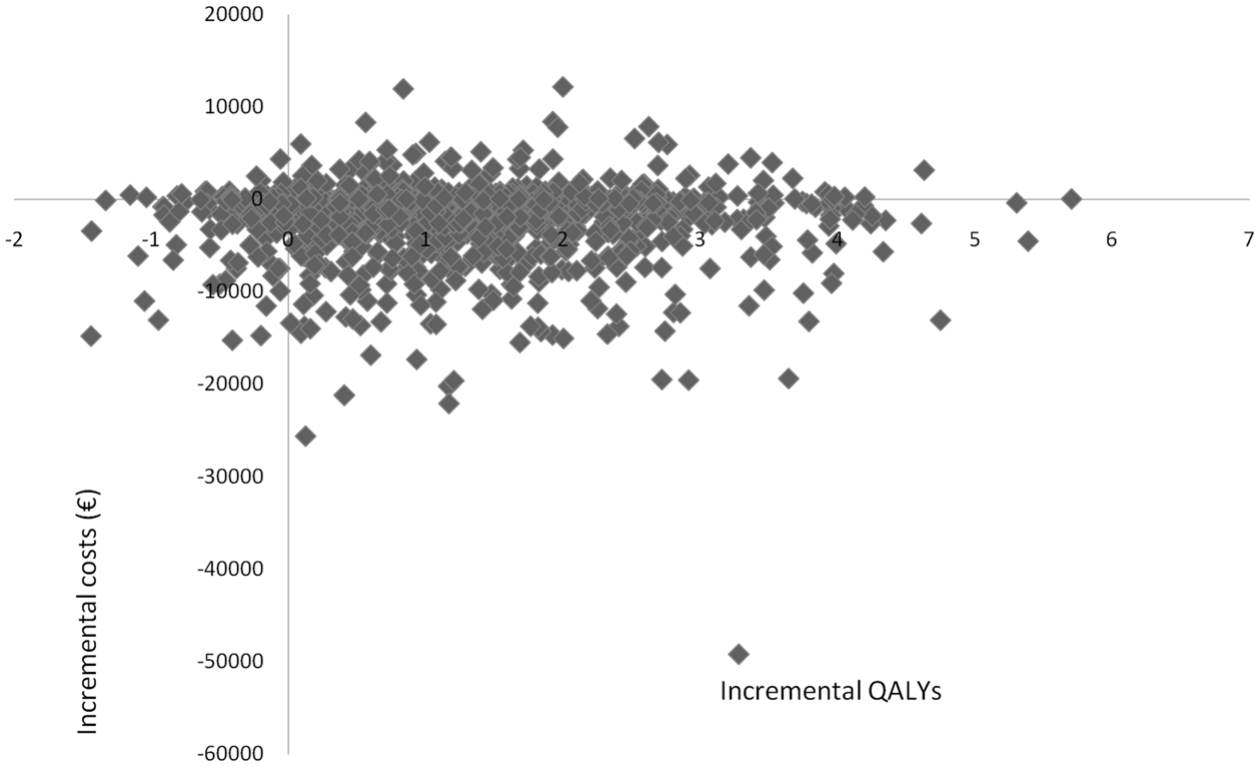
Cost-effectiveness plane showing 1000 replications from a distribution of cost and quality-adjusted life year (QALY) differences (angiotensin converting enzyme inhibitor vs microalbuminuria screening).

Figure 2 shows the cost-effectiveness acceptability curve, which considers uncertainty in cost-effectiveness. The probability of savings of the “treat all” strategy compared to screening for microalbuminuria is 70% (see also figure 3 for the scatterplot).

## Discussion

This modeling study shows that treating all patients with type 2 diabetes with ACE inhibitors (and more expensive ARBs in the event of cough) immediately after diagnosis is cost-effective and even reduces health care expenditures in the Dutch setting. The results were robust to a variety of different assumptions of uncertainty.

Although a significant number of newly diagnosed type 2 patients may receive blood pressure medications, there is no evidence to date that these patients are primarily prescribed an ACE inhibitor, which underlines the significance of this analysis. Still, our model is far from being perfect, but in modeling studies this is rarely the case due to constraints of resources, time, and information availability.

In the present study, savings by treating all diabetic patients with ACE inhibitors may even be underestimated for several reasons. First, we did not model that ACE inhibitors and ARBs reduce the risk for cardiovascular events [57], which would lead to additional savings. Second, Second, we did not consider real-world compliance with ACE inhibitor therapy due to a lack of data. In the real world some patients discontinue ACE inhibitor therapy and thus do not incur any drug cost. On the other hand, the model considered trial-based compliance on the effect side, as the rate of compliance is implicitly incorporated in clinical trial results, i.e., efficacy data refer both to adherers and non-adherers. For this reason the Markov model includes patients who discontinue ACE inhibitor treatment in the ACE inhibitor arm.

Third, the screening costs considered for microalbuminuria screening are based on one annual test only. In contrast, considering recommended screening procedures from the PREVEND IT study [58], [59] as a basis would lead to a fundamental increase of screening costs as a spot-urine sample (either the first-morning void or at the time of the visit to the medical office) was used as a pre-screening. Patients whose urine is tested positive should have their 24-h urine samples tested repeatedly afterwards [60].

Forth, as this study is based on a cohort simulation it uses data on the population mean. In contrast, a patient-level simulation would account for the fact that some individuals may stay in more than 2 stages in a year, although this is rarely the case. In any case, if patients progressed more rapidly (had higher risk), then ACE inhibitor treatment could lead to an even larger absolute risk reduction and therefore larger savings.

Finally, costs of dialysis treatment will likely continue to rise in the future, thus increasing the potential for savings by preventing ESRD. Dialysis costs have increased within the last years [12] and we expect this trend to continue due to stricter regulations concerning dialysis safety, technological advancement of dialysis machines, and better-tolerated dialysis solutions. Further limitations of the model relate to the data sources.

First, the model uses some epidemiological data from Western countries other than the Netherlands. For example, we used a Finnish study [28] as the source of the distribution of health states at the time of diagnosis. However, changing the initial distribution of health states had little impact on the outcome.

Second, transition rates from macroalbuminuria to ESRD with and without ACE inhibitors were not available for patients with type 2 diabetes. Therefore, we used a randomized controlled trial in patients with type 1 diabetes as the source [15].

Third, the standardized mortality ratio (SMR) we applied to diabetic patients without ESRD [1] includes patients with ESRD. Excluding these patients would lower the SMR to a minor degree as less than 2% of the Dutch diabetic population receives renal replacement therapy [35].

Forth, we assumed that the SMR is the same for patients with normo-, micro-, and macroalbuminuria as there are no valid data showing that a significant difference exists. The slightly higher mortality ratio in microalbuminuric patients in the HOPE study (2000) [57] was most likely the result of prior cardiovascular events. There is no evidence in the literature that mortality rates increase only on the basis of the level of albumin in the urine. This is the same with the utilities, which are assumed to do not differ between different stages of albuminuria.

Finally, having microalbuminuria or macroalbuminuria might cause disutility due to anxiety. However, standard preference measures such as the SG or the TTO method are not able to capture anxiety over future events as both evaluation methods assume a constant health state over the remaining period of life.

Compared to previous cost-effectiveness models, which were conducted by Golan et al. (1999) [22] and Rosen et al. (2005) [23] based on U. S. data, a much broader evidence base for the transition between normo- to microalbuminuria and micro- to macroalbuminuria was included in the present study. In addition, we considered that patients who are noncompliant with ACE inhibitors due to cough may receive more expensive ARBs, as similarly done for the German setting [24]. The fact that a small proportion of patients on ARBs (3.2%) also develop cough [17] and thus may discontinue treatment was disregarded. The reason for the exclusion is that noncompliance with treatment is already incorporated in the relative risk of treatment (thus lowering the relative risk), as in RCTs a certain proportion of patients discontinued treatment. In contrast to the previous models mentioned above we additionally conducted the analysis including an ARB for the entire patient population in need of treatment. This was done as some studies question that ARBs are not only a more expensive, but also a more effective alternative compared to ACE inhibitors. As the breakeven point is higher than the annual treatment costs of the ARB therapy this strategy must be considered cost-effective. However, we assumed equal effectiveness of all ACE inhibitors and ARBs, as meta-analyses do not suggest any independent effect of single renin-angiotensin-system agents [61], [30]. For instance, an ARB as an equivalent but more expensive alternative should only be prescribed in case of a contraindication (e.g. dry cough associated with ACE inhibitor treatment).

Still, similar to Adarkwah et al. (2010) [24] our model shows that treating all newly diagnosed type 2 diabetic patients with ACE inhibitors saves costs. The probability of savings is higher in Germany than in the Netherlands (89% vs. 70%). Reasons for this difference are not obvious as, e.g., costs of screening, ACE inhibitor treatment, and ESRD are quite similar. In contrast to Rosen et al. (2005) [23], we did not consider the preventive effect of ACE inhibitors on cardiovascular outcomes, which would have increased savings. An important reason for the large savings potential in the Netherlands is the low price of enalapril, which has substantially decreased during the last few years [46], [62]. The most ARBs are still protected by patent. Irbesartan, which we included in our study, is protected until March 2012. Assuming that prices of ARBs will decline after expiration of the patent protection would further strengthen our conclusion.

For patients with type 2 diabetes treatment with an ACE inhibitor to prevent the occurrence or progression of diabetic kidney disease is highly cost-effective. Current national guidelines, which do not even consistently recommend an ACE inhibitor for patients with microalbuminuria need to be reconsidered. Still, it is unclear whether a societal perspective leads to smaller or larger savings than a health care perspective. For instance, ACE inhibitor treatment avoids productivity loss due to renal failure and copayments for the treatment of renal failure, but drug copayments lead to additional costs.
